# Frontal Lobe Cysticercosis

**DOI:** 10.7759/cureus.82321

**Published:** 2025-04-15

**Authors:** Ranjan K Singh

**Affiliations:** 1 Internal Medicine, Jai Prakash Narayan Hospital, Gaya, IND

**Keywords:** albendazole, attention-deficit, cysticercosis, frontal lobe, taenia sodium

## Abstract

Cysticercosis, a brain infection caused by the parasite Taenia solium, presents considerable health challenges, especially in tropical and developing areas, where it contributes significantly to the incidence of epilepsy. The lifecycle of this parasite involves humans as the primary hosts and pigs as the intermediate hosts. In this case study, a 12-year-old girl exhibited unusual symptoms, such as fainting episodes and a decline in academic performance. Although she adhered to a vegetarian diet, her contact with pigs resulted in an infection, confirmed by a CT scan that revealed a cyst in the right frontal lobe containing a hyperdense scolex. The treatment regimen included valproic acid, prednisolone, and albendazole, which successfully resolved her seizures over a follow-up period of 24 months. This case underscores the diagnostic challenges associated with cysticercosis and highlights the necessity of identifying atypical cognitive symptoms related to this infection, particularly in populations at risk of exposure.

## Introduction

Cysticercosis is the most common parasitic infection of the brain in tropical and developing countries and can cause epileptic seizures. Taenia solium completes its lifecycle in two hosts: humans (definitive host), and pigs (intermediate host). Humans harbor the adult tapeworm, while pigs carry the larval form (cysticercus cellulosae), which consist of fluid-filled vesicles containing a tapeworm head (scolex) and are typically ingested through contaminated pork by humans. Once ingested, the scolex attaches to the intestinal wall, maturing into a tapeworm. Gravid proglottids and eggs are excreted with feces, facilitating transmission. Humans can sometimes act as intermediate hosts, getting infected with larva or eggs from T. solium. These ova invade the intestinal wall into blood vessels and get established in the brain, eyes, and subcutaneous tissue [1].

In endemic regions, particularly tropical and subtropical areas, T. solium is a significant health concern, accounting for approximately 30% of epilepsy cases and correlating with up to 70% of cases in some studies [2,3]. Although T. solium is not endemic in the United States, cases can occur primarily due to immigration, with a prevalence of 0.2 to 0.6 per 1000 000 people in certain western states [1].

Clinically, T. solium infection can lead to neurological symptoms such as seizures and headaches, which arise when the larvae invade the central nervous system. Peak incidence occurs between 20 and 50 years of age, while reports of infection in toddlers and children with atypical presentations are uncommon [4].

## Case presentation

**Figure 1:**
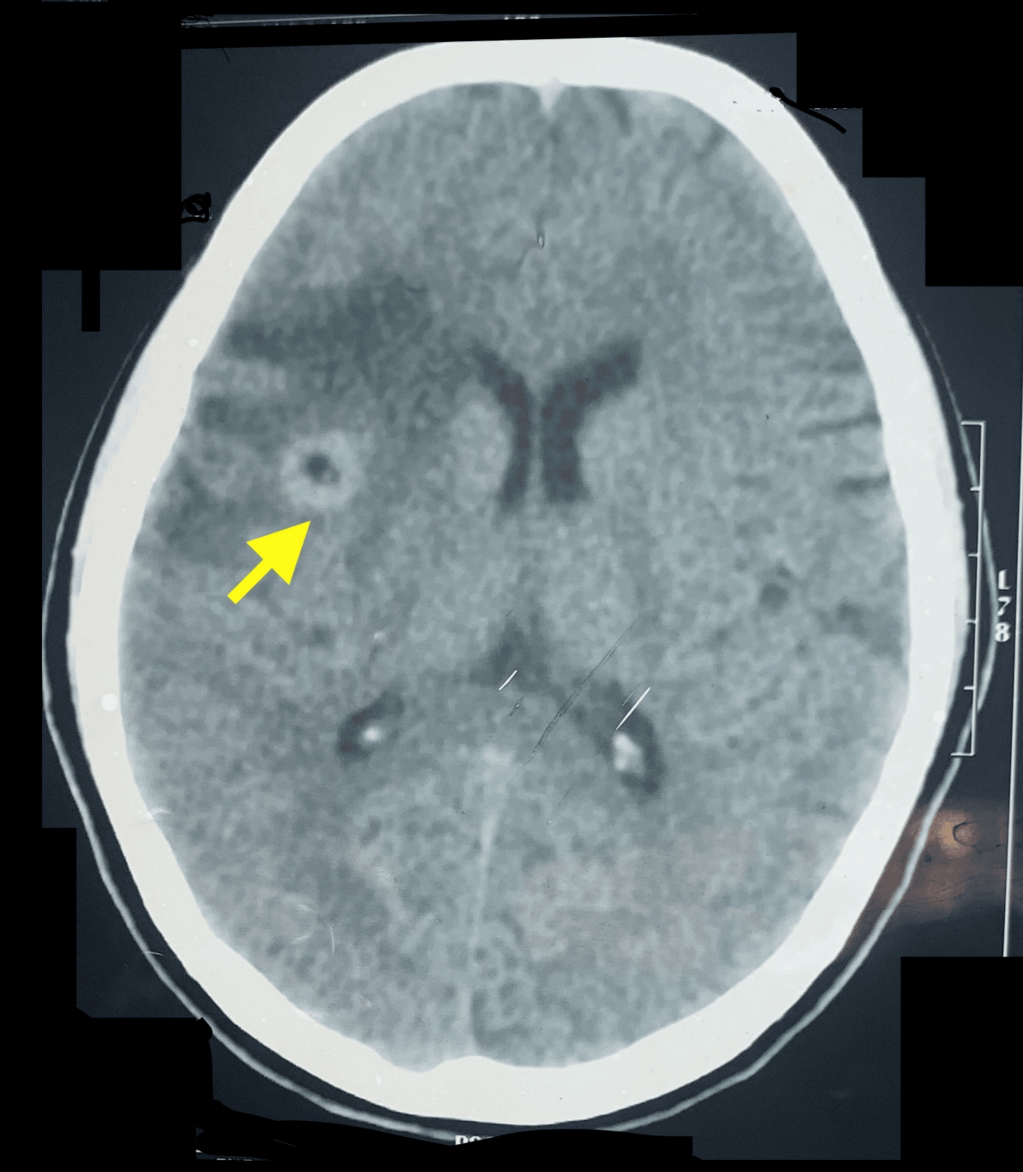
An unenhanced CT scan shows a single parenchymal cyst in the right frontal lobe of the cerebral cortex. Inside the cyst, there was a hyperdense dot with a ' hole-with-dot' sign; extensive edema around the cyst was also noted.

A 12-year-old girl had been suffering from repeated instances of fainting that lasted from a few seconds to a minute with a frequency of three to four times during the past three months. Her parents recently noted a decline in her academic performance. Although the family followed a vegetarian diet, there were pigs living nearby. A clinical examination revealed that her neurological functions were intact. Attention deficits were noted resulting in a score of 8 on the scale used in the Diagnostic and Statistical Manual of Mental Disorders, Fifth Edition (DSM-5). Blood tests showed a hemoglobin level of 11.5 gm/dl, a white blood cell count of 108x10^9/L with 70% neutrophils and 10% eosinophils, and an erythrocyte sedimentation rate (ESR) of 30 mm in the first hour. An unenhanced computed tomography (CT) scan of the brain identified a lesion in the right frontal lobe, characterized by a single cyst containing a hyperdense dot (scolex) at its center, along with surrounding edema (Figure 1).

The imaging findings led to a diagnosis of cysticercosis. The patient was prescribed valproic acid at a dosage of 200 mg orally twice daily, along with prednisolone at 5 mg once daily and albendazole at a dose of 15 mg per kg of body weight for 14 days. Following this treatment, the prednisolone dosage was gradually reduced and ultimately discontinued after one week. Valproic acid was continued for three months before being tapered off over the next three months. Patient’s white blood cell count and serum alanine aminotransferase were monitored.

**Figure 2:**
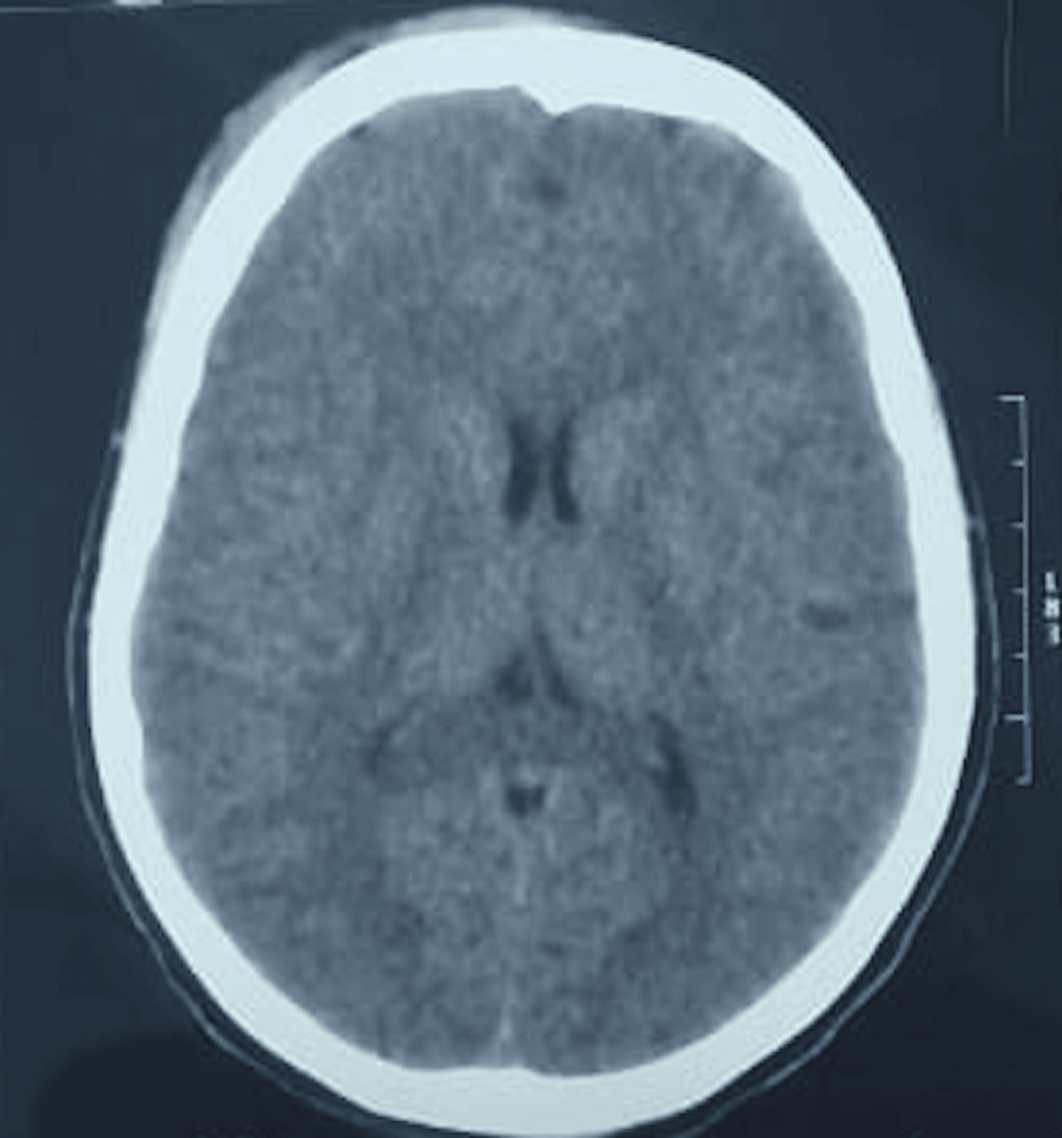
A CT scan of brain shows no residual parenchymal lesion.

Before the initiation of treatment, the patient suffered from seizures occurring three to four times a day, with each episode lasting around one minute. However, there was a steady decline in the frequency of these seizures, and by the 10th day of treatment, all seizures had completely stopped. After three months of treatment, the cystic lesion was resolved on imaging, and the patient exhibited no signs of attention deficits or seizure activity. A follow-up period of 24 months included regular comprehensive clinical evaluations every three months to monitor for any neurological symptoms. The patient's epileptic seizures, which had decreased during the initial treatment phase, did not reappear throughout the 24-month follow-up. Additionally, a CT scan of the brain conducted at that time showed no residual lesions or calcifications (Figure 2).

## Discussion

An electroencephalogram (EEG) was not deemed necessary in this case due to the CT finding providing definitive evidence of cysticercosis, which is a known cause of epileptic seizures and other neurological symptoms. Neuroimaging is an established modality for the diagnosis of neurocysticercosis. The definitive identification of a scolex within a cystic lesion on a CT scan, appearing as a hyperdense dot, confirms the absolute diagnostic criteria of cysticercosis. Del Brutto et al. [5] proposed a diagnostic criterion for neurocysticercosis that is widely acknowledged. WHO’s recent guidelines on the management of Taenia solium neurocysticercosis suggests magnetic resonance imaging (MRI) is the tool of choice for the diagnosis of neurocysticercosis; CT scan should be used if MRI is not available or contraindicated; however, CT is the tool of choice for detection of small calcified lesion [6].

The clinical presentation of a single neurocysticercosis can include neurological symptoms. A parenchymal cyst in the brain typically presents with epileptic seizures, often starting as simple partial seizures before progressing to generalized seizures. Cognitive impairments and dementia are also reported manifestations in adults. While the occurrence of attention deficits in children with neurocysticercosis is rarely reported, the presence of a cystic lesion accompanied by significant edema in the frontal lobe can cause this due to dysfunction of the frontostriatal track [7]. The clinical symptoms, such as intermittent short episodes of loss of consciousness and a noticeable decrease in academic performance, highlight the atypical presentation of a frontal lobe cyst, which can be associated with cognitive impairments and seizure activity.

There are differing opinions on the use of antiparasitic medication (albendazole) and steroids in the management of single neurocysticercosis cases. Nevertheless, a dosage of 15 mg/kg of body weight of albendazole for a duration of 10 to 14 days is recommended for patients with a single viable cyst [8]. Albendazole, in combination with corticosteroids, should be given to individuals with symptomatic neurocysticercosis or a single enhancing lesion for better outcomes in terms of cyst resolution and seizure control. Conversely, it should be avoided in calcific cysticercosis [6]. In cases where cerebral edema is present, steroids should be administered at minimal doses for the shortest necessary duration [8]. 

A close differential diagnosis in developing countries is tuberculoma, especially in the Indian subcontinent, which is hard to distinguish from neurocysticercosis at times. Tuberculomas can be seen in a plain CT scan as hypodense, isodense, or hyperdense lesions with surrounding oedema, while a cystic lesion and a scolex visible inside the cyst are characteristic of cysticercosis [9].

## Conclusions

This case study emphasizes the complexities involved in diagnosing frontal lobe cysticercosis in a 12-year-old girl who exhibited atypical symptoms, including attention deficits, in addition to the more common seizures. An unenhanced CT scan revealed a characteristic cyst with a hyperdense scolex, confirming this diagnosis. 

Effective treatment with valproic acid, prednisolone, and albendazole led to the resolution of seizures without any residual lesion or calcification on imaging over a 24-month follow-up, reinforcing the importance of a tailored treatment approach. This case emphasizes the need for awareness regarding cysticercosis, particularly in endemic regions or in populations with potential exposures, as cognitive symptoms can be significant and may mimic other neurological disorders.

## References

[1] Garcia HH, Nash TE, Del Brutto OH (2014). Clinical symptoms, diagnosis, and treatment of neurocysticercosis. Lancet Neurol.

[2] Bustos J, Gonzales I, Saavedra H, Handali S, Garcia HH (2021). Neurocysticercosis. A frequent cause of seizures, epilepsy, and other neurological morbidity in most of the world. J Neurol Sci.

[3] (2025). Taeniasis/cysticercosis. https://www.who.int/news-room/fact-sheets/detail/taeniasis-cysticercosis.

[4] Del Brutto OH (2013). Neurocysticercosis in infants and toddlers: report of seven cases and review of published patients. Pediatr Neurol.

[5] Del Brutto OH, Nash TE, White AC Jr (2017). Revised diagnostic criteria for neurocysticercosis. J Neurol Sci.

[6] (2025). Guidelines on management of Taenia solium neurocysticercosis. http://www.who.int/publications/i/item/9789240032231.

[7] Barboza M, Sepúlveda S, Montalvo D (2007). Frontal neurocysticercosis and attention deficit. Colomb Med.

[8] Sankhyan N, Kadwa RA, Kamate M (2021). Management of neurocysticercosis in children: Association of Child Neurology consensus guidelines. Indian Pediatr.

[9] (2025). Tuberculoma. http://radiopaedia.org/articles/24731.

